# Identification Exploring the Mechanism and Clinical Validation of Mitochondrial Dynamics-Related Genes in Membranous Nephropathy Based on Mendelian Randomization Study and Bioinformatics Analysis

**DOI:** 10.3390/biomedicines13061489

**Published:** 2025-06-17

**Authors:** Qiuyuan Shao, Nan Li, Huimin Qiu, Min Zhao, Chunming Jiang, Cheng Wan

**Affiliations:** Department of Nephrology, Nanjing Drum Tower Hospital, The Affiliated Hospital of Nanjing University Medical School, Nanjing 210023, China; qiuyshao@163.com (Q.S.); linannj2012@163.com (N.L.); min999746@163.com (H.Q.); zhaomin20081988@126.com (M.Z.)

**Keywords:** membranous nephropathy, mitochondrial dynamics, mendelian randomization, machine learning, single-cell analysis

## Abstract

**Background:** Membranous nephropathy (MN), a prevalent glomerular disorder, remains poorly understood in terms of its association with mitochondrial dynamics (MD). This study investigated the mechanistic involvement of mitochondrial dynamics-related genes (MDGs) in the pathogenesis of MN. **Methods:** Comprehensive bioinformatics analyses—encompassing Mendelian randomization, machine-learning algorithms, and single-cell RNA sequencing (scRNA-seq)—were employed to interrogate transcriptomic datasets (GSE200828, GSE73953, and GSE241302). Core MDGs were further validated using reverse-transcription quantitative polymerase chain reaction (RT-qPCR). **Results:** Four key MDGs—RTTN, MYO9A, USP40, and NFKBIZ—emerged as critical determinants, predominantly enriched in olfactory transduction pathways. A nomogram model exhibited exceptional diagnostic performance (area under the curve [AUC] = 1). Seventeen immune cell subsets, including regulatory T cells and activated dendritic cells, demonstrated significant differential infiltration in MN. Regulatory network analyses revealed ATF2 co-regulation mediated by RTTN and MYO9A, along with RTTN-driven modulation of ELOA-AS1 via hsa-mir-431-5p. scRNA-seq analysis identified mesenchymal–epithelial transitioning cells as key contributors, with pseudotime trajectory mapping indicating distinct temporal expression profiles: NFKBIZ (initial upregulation followed by decline), USP40 (gradual fluctuation), and RTTN (persistently low expression). RT-qPCR results corroborated a significant downregulation of all four genes in MN samples compared to controls (*p* < 0.05). **Conclusions:** These findings elucidate the molecular underpinnings of MDG-mediated mechanisms in MN, revealing novel diagnostic biomarkers and therapeutic targets. The data underscore the interplay between mitochondrial dynamics and immune dysregulation in MN progression, providing a foundation for precision medicine strategies.

## 1. Introduction

Membranous nephropathy (MN) is a prominent glomerular pathology characterized by the deposition of subepithelial immune complexes along the glomerular basement membrane, leading to the clinical manifestation of nephrotic syndrome [1]. Epidemiological studies indicate that the global incidence of membranous nephropathy (MN) is approximately 8–10 cases per million population. The incidence is marginally higher in the United States, reaching 12 cases per million population [2]. In China, MN accounts for over 20% of primary glomerular diseases, with primary MN constituting approximately 80% of all MN cases [2,3]. Among White adults, MN represents the most common etiology of primary nephrotic syndrome [3]. The clinical trajectory of MN exhibits considerable heterogeneity: while spontaneous remission occurs in roughly one-third of cases, another third maintain stable renal function despite persistent proteinuria. The remaining third face a substantial risk of progression to end-stage renal disease in the absence of timely diagnosis and intervention. Existing therapeutic regimens—centered on immunosuppressive therapy and supportive management—often fail to prevent relapse, highlighting a critical need for novel and more effective treatment strategies [4,5]. Elucidating the molecular mechanisms underlying MN is therefore imperative for the discovery of reliable diagnostic biomarkers and the development of targeted therapeutic interventions to improve clinical outcomes.

Mitochondrial dynamics (MD), encompassing mitochondrial fusion, fission, and turnover processes, are essential for maintaining organellar integrity and bioenergetic function. These dynamic processes are integral to ATP generation and exert regulatory influence over key cellular activities, including apoptosis and intracellular signaling pathways [6,7]. Disruptions in MD have been implicated in the pathophysiology of MN, where impaired mitochondrial function may amplify oxidative stress and inflammatory responses, thereby exacerbating renal injury [8,9]. Notably, elevated expression levels of mitochondrial dynamin-related protein 1 (Drp1) and fission protein 1 (Fis1) have been observed in pediatric patients with MN-associated nephrotic syndrome, particularly among those diagnosed with MN [10], suggesting a mechanistic role for aberrant MD in disease initiation and progression. Despite these findings, comprehensive investigations into MD-related genes (MDGs) within the context of MN remain limited. Advancing research in this domain holds the potential to unravel the specific contributions of MDGs to MN pathogenesis and to identify novel molecular targets for early diagnostic and therapeutic interventions.

RNA sequencing (RNA-seq) enables a comprehensive assessment of transcriptional activity across the entire genome within cells or tissues, offering critical insights into the molecular mechanisms driving disease onset and progression [11]. Mendelian randomization (MR), a cutting-edge analytical framework, leverages genetic variants as instrumental variables (IVs) to infer causal relationships between exposures and outcomes, thereby mitigating confounding biases inherent in traditional observational studies [12,13]. Advances in single-cell technologies, particularly single-cell RNA-seq (scRNA-seq), have revolutionized the understanding of cellular heterogeneity by facilitating high-resolution profiling of gene expression at the single-cell level. This technology enables the identification of distinct cellular states and intercellular interactions within complex tissue environments [14,15]. In the context of MN, the integration of RNA-seq, MR, and scRNA-seq provides a multidimensional framework—from transcriptional profiling and causal inference to cellular-level characterization—allowing for a more comprehensive elucidation of the disease’s molecular underpinnings.

This study is designed to systematically investigate the mechanistic roles of macrophage-derived genes (MDGs) in membranous nephropathy (MN) through integrated bioinformatics strategies, including Mendelian randomization (MR), single-cell transcriptomic analysis, and machine learning. The workflow will initially involve curating MN-associated transcriptomic datasets from publicly available repositories, followed by a tripartite analytical framework combining MR analysis, machine-learning algorithms, and experimental expression validation to identify MD-related hub genes. The immunological relevance and regulatory networks associated with these candidate genes were further delineated through immune cell infiltration analysis and molecular interaction mapping. Additionally, scRNA-seq was utilized to characterize the cell-type-specific expression and spatial distribution patterns of the identified MDGs in MN-affected tissues. This integrative approach advances our mechanistic understanding of MN pathophysiology and lays the groundwork for the development of targeted therapeutic strategies.

## 2. Materials and Methods

### 2.1. Data Collection

Transcriptomic datasets associated with MN—GSE200828 (GPL19983), GSE73953 (GPL4133), and GSE108109 (GPL19983)—along with the scRNA-seq dataset GSE241302 were retrieved from the Gene Expression Omnibus (GEO) repository (https://www.ncbi.nlm.nih.gov/geo/, accessed on 20 July 2024). GSE200828 comprises 57 glomerular biopsy samples, including 51 from patients with MN and 6 from healthy controls (CON group) [16]. GSE73953 includes 10 peripheral blood samples, obtained from 8 patients with MN and 2 healthy individuals [17], while GSE108109 contains glomerular biopsy tissues from 44 patients with MN and 6 healthy controls [17]. The single-cell dataset GSE241302 encompasses four kidney tissue samples—three from patients with MN and one from a healthy control) [18]. A total of 23 MDGs were curated from the Human MitoCarta 3.0 database (http://www.broadinstitute.org/mitocarta, accessed on 20 July 2024) [19].

### 2.2. Differential Expression Analysis

Differential expression analysis was conducted using the “limma” package (v3.54.1) [20], applying thresholds of |log2 fold change (FC)| > 0.5 and *p* < 0.05. This analysis yielded differentially expressed genes (DEGs): DEGs1 for GSE200828 and DEGs2 for GSE73953. Visualization of DEGs1 and DEGs2 was performed using volcano plots and heatmaps generated via the “ggplot2” (v3.4.1) and “ComplexHeatmap” (v2.14.0) [21,22] packages, respectively. The “ggvenn” package (v1.7.3) [23] was employed to identify overlapping differentially expressed genes by intersecting upregulated DEGs1 with upregulated DEGs2, and downregulated DEGs1 with downregulated DEGs2. The merged upregulated and downregulated intersected genes were collectively designated as DEGs.

### 2.3. Weighted Gene Co-Expression Network (WGCNA) Analysis

To quantify MDG-related activity across samples, the single-sample gene set enrichment analysis (ssGSEA) algorithm from the “GSVA” package (v1.46.0) [24] was utilized to compute ssGSEA scores for all samples in GSE200828. Differences in ssGSEA scores between MN and CON groups were assessed using the Wilcoxon rank-sum test (*p* < 0.05). WGCNA was subsequently conducted using the “WGCNA” package (v1.73) [25], with ssGSEA scores treated as the phenotypic trait. Sample clustering and outlier exclusion were executed via the hclust function. The optimal soft-thresholding power was determined using the pickSoftThreshold function by optimizing the scale-free topology criterion (target R^2^ and approximate zero connectivity). Module clustering was performed using the hierarchicalCluster function. Correlations between module eigengenes and phenotypic traits were computed to identify associations, and two modules exhibiting the strongest positive and negative correlations with ssGSEA scores were selected as key modules. The intersecting genes between these key modules and previously defined DEGs were identified using the “ggvenn” package (v1.7.3) and designated as MD-DEGs.

### 2.4. Identification and Functional Analysis of MD-DEGs

Gene Ontology (GO) and Kyoto Encyclopedia of Genes and Genomes (KEGG) enrichment analyses were performed using the “clusterProfiler” package (v0.6.8) [26] to elucidate the biological functions and pathways associated with the MD-related differentially expressed genes (MD-DEGs). A significance threshold of *p* < 0.05 was applied to identify enriched terms. To investigate protein-level interactions among mitochondrial dynamic-differentially expressed genes (MD-DEGs), a protein–protein interaction (PPI) network was constructed using the STRING database (https://cn.string-db.org), with a minimum required interaction score set at 0.4.

### 2.5. Selection of Single Nucleotide Polymorphisms (SNPs)

To explore potential causal relationships between MD-DEGs and MN, MR analysis was conducted. Expression quantitative trait loci (eQTL) data for MD-DEGs were retrieved from the Integrative Epidemiology Unit (IEU) Open GWAS database (https://gwas.mrcieu.ac.uk/), and genome-wide association study (GWAS) summary statistics for MN were obtained under the outcome ID ebi-a-GCST010005, comprising 5,327,688 single nucleotide polymorphisms (SNPs) derived from 7979 individuals (2150 MN cases and 5829 controls). In this analysis, MD-DEGs were treated as exposure variables and MN as the outcome. The validity of MR was assessed based on three core assumptions: (1) a robust and statistically significant association between SNPs and exposure; (2) the independence of SNPs from potential confounding factors; (3) the exclusive influence of SNPs on the outcome via the exposure pathway, without pleiotropic effects. SNPs associated with exposure variables at genome-wide significance (*p* < 5 × 10^−8^) were extracted using the extract_instruments function from the TwoSampleMR package (v0.6.8) [27]. To minimize the risk of linkage disequilibrium (LD), clumping was performed with parameters set to clump = TRUE, r^2^ = 0.001, and a window size of 500 kb. Instrument strength was evaluated using the F-statistic, with SNPs deemed sufficiently strong when F > 10, calculated as F = (β^2^)/(SE^2^).

### 2.6. Mendelian Randomization (MR) Analysis

The harmonise_data function from the “TwoSampleMR” package (v0.6.8) was employed to align effect alleles and standardize effect sizes across datasets. MR was conducted using a suite of analytical methods, including MR-Egger [28], weighted median [29], inverse variance weighted (IVW) [30], simple mode, and weighted mode [27,31]. Among these, IVW was prioritized as the principal method due to its superior statistical power in detecting causal relationships. An odds ratio (OR) exceeding 1 was interpreted as indicative of a risk factor, whereas an OR below 1 suggested a protective effect.

To evaluate the robustness and reliability of causal inferences, multiple sensitivity analyses were conducted. Cochran’s Q test was first applied to detect heterogeneity among IVs; a *p*-value greater than 0.05 was considered indicative of homogeneity. Horizontal pleiotropy was assessed using the mr_pleiotropy_test function, with *p* > 0.05 indicating the absence of directional pleiotropy. In addition, a leave-one-out (LOO) analysis was performed, iteratively excluding individual SNPs to evaluate their influence on overall causal estimates. To further validate the causal direction, the Steiger directionality test was employed. Only those genes for which the test confirmed the correct causal direction (*p* < 0.05 and direction = TRUE) were retained as candidate characterization genes for downstream analyses. All data utilized were accessed on 3 September 2024. The GWAS dataset for the outcome variable (ebi-a-GCST010005) met the essential criteria, including a sufficiently large sample size (case count > 5000) and direct use of original GWAS-derived SNP associations, thereby minimizing bias from proxy variants. Dataset sources included high-quality repositories such as the UK Biobank, FinnGen, and the GWAS Catalog, ensuring the methodological rigor and validity of the analysis.

### 2.7. Identification and Analysis of Key Genes

To identify definitive characterization genes, multiple machine-learning algorithms were applied. First, least absolute shrinkage and selection operator (LASSO) regression analysis was performed on GSE200828 using the “glmnet” package (v4.1.8) [32] to yield candidate characterization genes 1. A random forest (RF) model was subsequently constructed using the “randomForest” package (v4.7-1.2) to derive candidate characterization genes 2 [33]. Additionally, the Boruta algorithm was implemented via the “Boruta” package (v8.0.0) to obtain candidate characterization genes 3 [34]. The intersection of gene sets derived from these three models was determined using the “ggvenn” package (v1.7.3), and the overlapping genes were defined as final characterization genes. Expression validation of these genes was conducted across three independent datasets: GSE200828, GSE73953, and GSE108109. Genes demonstrating statistically significant differential expression (*p* < 0.05) between MN and control groups, along with consistent expression trends across all datasets, were designated as key genes.

### 2.8. Construction of Nomogram

To forecast the likelihood of having MN, the key genes were integrated for prediction purposes and a nomogram was developed. Firstly, five-fold cross-validation was first performed using the “rms” package (v 6.8-1) [34] to assess model performance. Stratified sampling was conducted via the “createFolds” function to ensure that the sample distribution in each fold was similar to that in the original dataset. The model with the highest area under the curve (AUC) value was selected. Subsequently, a nomogram model was constructed through the “regplot” package (v 1.1) [35]. According to the nomogram, each key gene in the sample corresponds to a point, and the total point is obtained by adding up the points of all key genes, and a higher total point indicates a greater risk of developing MN. Finally, the receiver operating characteristic (ROC) curve for the nomogram was generated using the “pROC” package (v 1.18.5) [36], and the AUC value was computed to evaluate its capacity for accurate diagnosis of MN. An AUC > 0.7 was considered to indicate good performance.

### 2.9. Analysis of Correlation and Functional Resemblance Among Key Genes

To examine inter-gene relationships, Spearman correlation coefficients were computed among the key genes within MN samples of the GSE200828 dataset using the “psych” package (v4.1.8). Genes with absolute correlation coefficients (|cor|) > 0.3 and *p* < 0.05 were considered significantly correlated. Functional similarity among key genes was further evaluated using the “GOSemSim” package (v2.24.0) [37], providing insight into shared biological roles based on GO semantic similarity metrics.

### 2.10. Chromosome and Subcellular Localization Analysis

The chromosomal distribution of the key genes was visualized using the “RCircos” package (v1.2.2) [38], offering a graphical overview of their genomic loci. To identify subcellular localization patterns, mRNA sequences of the key genes were retrieved from the National Center for Biotechnology Information (NCBI; https://www.ncbi.nlm.nih.gov) and analyzed using the mRNALocater database (http://bio-bigdata.cn/mRNALocater/), revealing the intracellular compartments where these genes predominantly function.

### 2.11. Gene Set Enrichment Analysis (GSEA)

GSEA was conducted to explore the biological functions and signaling pathways associated with the key genes. Genes were ranked by the log2 fold change (log2FC) between MN and control samples. The reference gene set used was c2.cp.kegg.v2023.1.Hs.symbols.gmt. Single-gene GSEA was performed using the “clusterProfiler” package (v4.2.2), and enrichment results were visualized with “ggplot2” (v3.4.1), applying a significance threshold of *p* < 0.05.

### 2.12. Immune Cell Infiltration and Immune Factor Correlation

To assess immune landscape alterations, the ssGSEA algorithm was applied to the GSE200828 dataset to compute enrichment scores for 28 immune cell types [39] across MN and CON samples [39]. Differences in immune cell infiltration were evaluated using the Wilcoxon rank-sum test. Immune cell types with statistically significant differences (*p* < 0.05) between groups were classified as differential immune cells. Correlation analysis among these immune cell subsets was subsequently performed using Spearman’s method via the “psych” package (v2.2.9) [40], with significance thresholds set at |correlation coefficient| > 0.3 and *p* < 0.05. To further investigate the immunological context of MN, associations between key genes and immunological factors—including immunostimulatory and immunosuppressive molecules, chemokines, and their receptors—were examined. Data on immune-related factors were obtained from the tumor–immune system interactions (TISIDB) database (http://cis.hku.hk/TISIDB/), and correlation analyses were performed using Spearman’s method through the “psych” package (v2.2.9), with criteria of |cor| > 0.3 and *p* < 0.05.

### 2.13. Construction of Regulatory Networks

To further elucidate the potential molecular regulatory mechanisms associated with the key genes, a transcription factor (TF)–gene regulatory network was constructed. TFs regulating the key genes were predicted using chromatin immunoprecipitation sequencing (ChIP-seq) data from the encyclopedia of DNA elements (ENCODE) database (https://www.encodeproject.org) via the NetworkAnalyst platform (https://www.networkanalyst.ca). The resulting regulatory interactions were visualized using Cytoscape software (v3.9.1) [41]. In parallel, a comprehensive long non-coding RNA (lncRNA)-microRNAs (miRNA)–mRNA regulatory network was established. mRNA sequences of the key genes were retrieved from the national center for biotechnology information (NCBI) database (https://www.ncbi.nlm.nih.gov), and their regulatory miRNAs were predicted using the miRDB database (http://mirdb.org/) via NetworkAnalyst. Subsequently, the StarBase database (http://starbase.sysu.edu.cn/) was used to identify lncRNAs targeting the corresponding miRNAs. The complete lncRNA–miRNA–mRNA interaction network was then graphically rendered using Cytoscape (v3.9.1).

### 2.14. Single-Cell Analysis

For scRNA-seq analysis, quality control (QC) was applied to the GSE241302 dataset by filtering cells based on the following criteria: nCount_RNA > 3, 200 < nFeature_RNA < 6000, and percent mitochondrial content (percent.mt) < 10%. The “Seurat” package (v5.0.1) [42] was used for downstream analysis. Highly variable genes (top 2000) were identified using the FindVariableFeatures function, and data normalization was performed via the NormalizeData and ScaleData functions. Principal component analysis (PCA) was conducted using the RunPCA function, and significant principal components were determined with the JackStraw and ElbowPlot functions. Unsupervised clustering was implemented using the FindNeighbors and FindClusters functions, while dimensionality reduction for visualization was performed using RunTSNE. To identify marker genes for each cluster, the FindAllMarkers function was applied with thresholds of |log2FC| > 1, expression ratio > 0.25, and adjusted *p* < 0.05. The top three genes with the highest fold changes were selected as marker genes. Differential expression analysis across cell types was also performed using the FindAllMarkers function with relaxed criteria (|log2FC| > 0.1, adjusted *p* < 0.05). Cluster annotations were manually curated based on known marker genes from the CellMarker database, allowing the accurate identification of cell types relevant to MN. The expression distribution of key genes across various cell types was examined, and cell populations exhibiting high expression levels of key genes were defined as key cells.

### 2.15. Cellular Communication and Pseudotime Analysis

To investigate intercellular communication within the GSE241302 dataset, the “CellChat” package (v1.6.1) [43] was employed to construct a cell–cell communication network. This enabled identification of ligand–receptor pairs mediating interactions between key cells and other cellular populations. To characterize dynamic changes in cell state over developmental time, pseudotime trajectory analysis was conducted using the “monocle” package (v2.22.0) [44], revealing lineage differentiation pathways and temporal expression dynamics within key cellular subpopulations.

### 2.16. Reverse-Transcription Quantitative Polymerase Chain Reaction (RT-qPCR)

Blood samples were collected from five individuals diagnosed with MN at Nanjing Drum Tower Hospital, the Affiliated Hospital of Nanjing University Medical School, along with samples from five healthy individuals, which served as controls. The study was conducted in accordance with the principles outlined in the Declaration of Helsinki (as revised in 2013) and was approved by the Ethics Committee of Nanjing Drum Tower Hospital, the Affiliated Hospital of Nanjing University Medical School (approval number: 2022-330-02, date: 7 July 2022). All samples were subjected to RT-qPCR analysis. The study protocol was approved by the Ethics Committee of Nanjing Drum Tower Hospital (approval number: 2022-330-04), and written informed consent was obtained from all participants. Total RNA was extracted from the ten samples using TRIzol reagent (Ambion, Austin, TX, USA) and subsequently reverse-transcribed into cDNA with the SureScript First-Strand cDNA Synthesis Kit (Servicebio, Wuhan, China). RT-qPCR was carried out using the 2 × Universal Blue SYBR Green qPCR Master Mix (Servicebio, Wuhan, China). Primer sequences are listed in Table S1, with GAPDH employed as the endogenous control. Gene expression levels were quantified using the 2^−ΔΔCt^ method [45]. Quantitative data from PCR assays were processed and visualized using GraphPad 10 software.

### 2.17. Statistical Analysis

Bioinformatic analyses were conducted using R software (v4.2.2). The Wilcoxon rank-sum test was applied to evaluate statistical differences between the MN and control groups in bioinformatics data. For RT-qPCR results, statistical significance was determined using the unpaired *t*-test, with *p* < 0.05 considered significant.

## 3. Results

### 3.1. Identification of MD-DEGs

**Figure 1:**
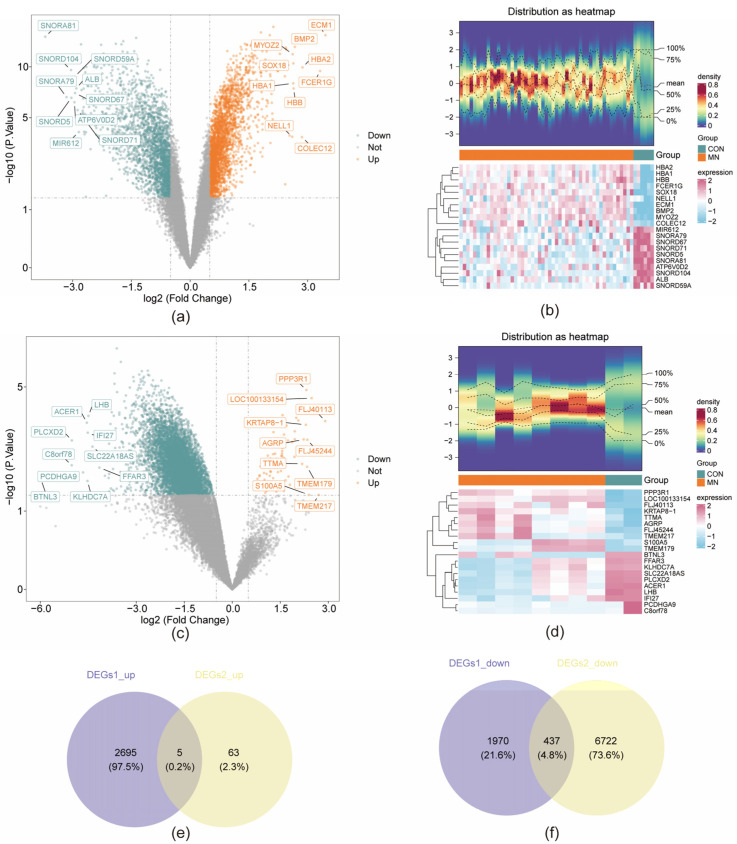
Identification of DEGs. (**a**) Volcano plot of DEGs1. Orange points represent upregulated genes, green points represent downregulated genes, and gray points represent genes with no significant difference under the threshold. (**b**) Expression heatmap of DEGs1. (**c**) Volcano plot of DEGs2. (**d**) Expression heatmap of DEGs2. (**e**) A total of 5 differentially upregulated intersection genes. (**f**) A total of 437 differentially downregulated intersection genes.

**Figure 2:**
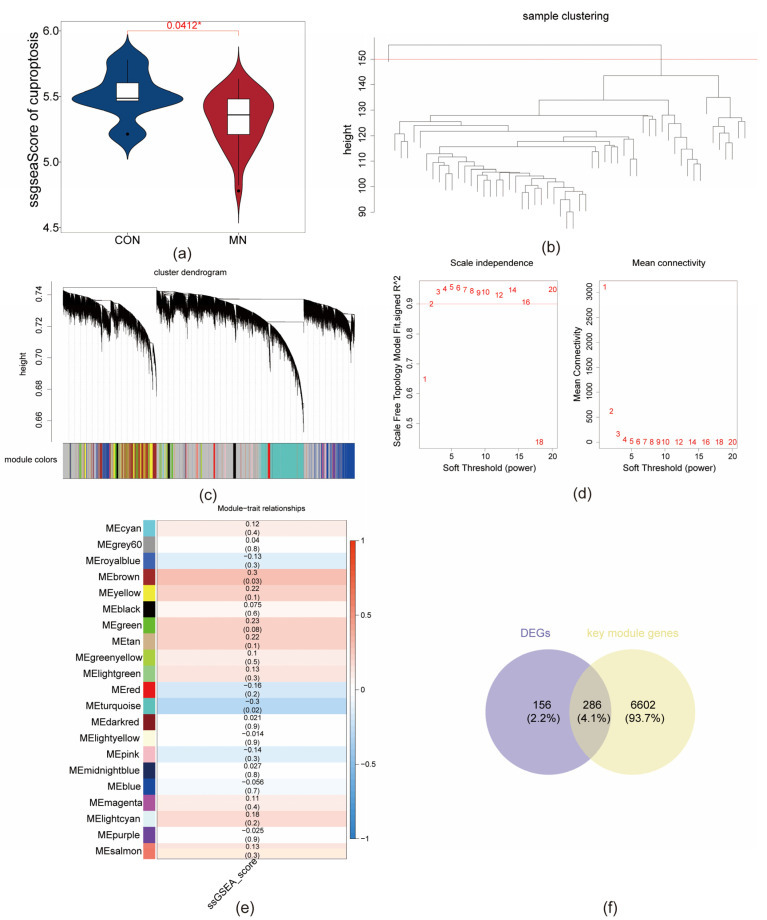
In total, 286 MD-DEGs were selected. (**a**) Box plot of MDGs score between MN and CON sample groups. *, *p* < 0.05 (**b**) Sample hierarchical clustering diagram. Each branch represents a sample, the red line represents the cutting line, and the vertical axis represents the Euclidean distance of sample expression. (**c**) Co-expression module identification. Different colors represent different modules. (**d**) Soft threshold selection. Left plot: Scale-free fitting index under different soft thresholds (*x*-axis); right plot: Network connectivity under different soft thresholds. The different numbers in the figure represent different soft thresholds. (**e**) Correlation heatmap between co-expression modules and MDGs scores. (**f**) Identification of 286 MD-DEGs.

A total of 5107 DEGs were identified in the GSE200828 dataset, comprising 2700 upregulated and 2407 downregulated genes (Figure 1a,b). In comparison, the GSE73953 dataset yielded 7227 DEGs, including 68 upregulated and 7159 downregulated genes (Figure 1c,d). Intersections were performed between the upregulated genes from both datasets and between the downregulated genes from both datasets. The resulting intersecting gene sets—5 upregulated and 437 downregulated—were combined to yield a final set of 442 overlapping DEGs (Figure 1e,f). ssGSEA scores were significantly lower in the MN group relative to the CON group (*p* < 0.05), indicating a potential association between ssGSEA scores and MN status for subsequent analyses (Figure 2a). To enhance analytical accuracy, hierarchical clustering was applied to the samples, and one outlier (GSM6044571) was excluded from further analysis (Figure 2b). GCNA identified an optimal soft-thresholding power (β) of 4 (Figure 2d), resulting in the construction of 21 gene co-expression modules (Figure 2c). Of these, the brown (cor = 0.3, *p* < 0.05) and turquoise (cor = −0.3, *p* < 0.05) modules demonstrated significant correlations with ssGSEA scores and were designated as key modules, encompassing a total of 6888 genes (Figure 2e). Intersecting these key module genes with the previously identified DEGs yielded 286 MD-DEGs (Figure 2e).

### 3.2. MD-DEGs Enriched Pathways and Protein Interactions Analysis

**Figure 3:**
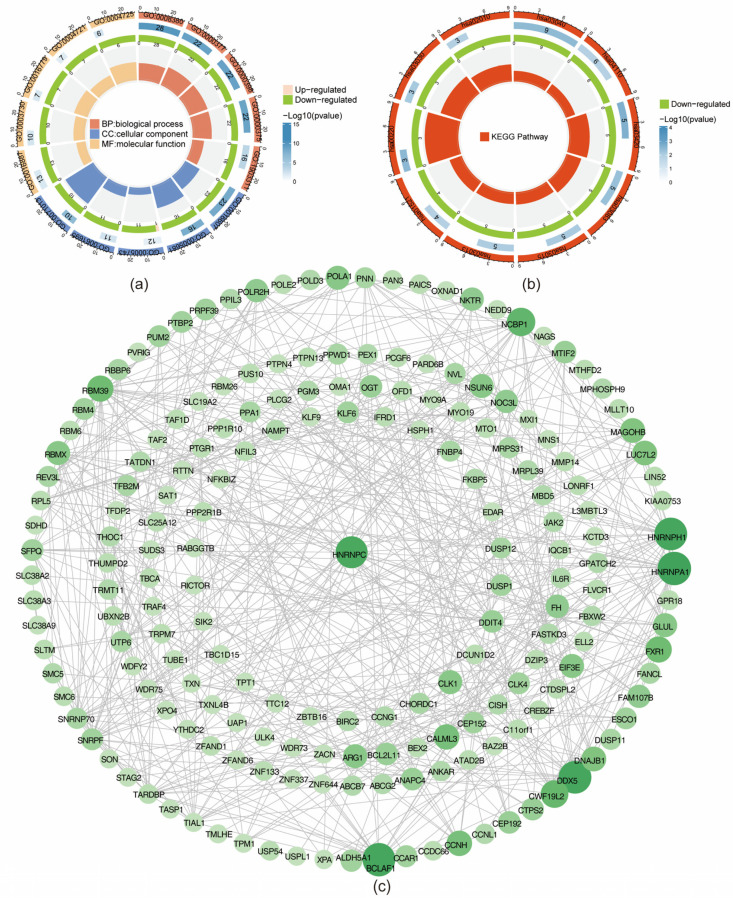
Protein interaction and functional prediction of MD-DEGs. (**a**) GO enrichment circle plot of MD-DEGs. (**b**) KEGG enrichment plot of MD-DEGs. (**c**) Protein interaction network diagram of MD-DEGs.

A total of 297 GO biological functions were enriched for MD-DEGs, comprising 211 biological processes (BP), 51 molecular functions (MF), 35 cellular components (CC), and 10 KEGG pathways. Functional enrichment analysis indicated that, within the BP category (Table S2), MD-DEGs were predominantly associated with RNA splicing, mRNA splicing, regulation of RNA splicing, regulation of mRNA metabolic processes, and ribonucleoprotein complex biogenesis. Within the CC category (Table S3), these genes were primarily linked to nuclear specks, spliceosomal complexes, the mitochondrial inner membrane, the catalytic step 2 spliceosome, and ribonucleoprotein granules. In the MF category (Table S4), significant associations were identified with phosphoric ester hydrolase activity, DNA-binding transcription repressor activity, phosphoprotein phosphatase activity, phosphatase binding, and magnesium ion binding (Figure 3a). KEGG pathway analysis revealed significant enrichment in spliceosome, cell cycle, nucleotide excision repair, polycomb repressive complex, and mRNA surveillance pathways (Figure 3b, Table S5). The PPI network derived from MD-DEGs comprised 491 interactions among 192 proteins. The most robust interactions included ABCB7–SLC19A2, ALDH5A1–NAGS, ANAPC4–PPWD1, ANKAR–WDR75, and ARG1–NAGS (Figure 3c).

### 3.3. MR Screening of Candidate Genes

MR analysis was conducted to identify MD-DEGs with a putative causal relationship to MN. A total of 286 MD-DEGs were evaluated to extract SNPs for subsequent MR analysis. Fourteen genes—USP40, ZFAND1, FNBP4, GPR18, TIGD7, NOC3L, WDR73, PPIL3, MYO9A, MTIF2, BAZ2B, NFKBIZ, OSGIN2, and RTTN—exhibited statistically significant causal associations with MN. Among them, eight genes (USP40, ZFAND1, FNBP4, GPR18, TIGD7, NOC3L, WDR73, and PPIL3) were identified as risk factors (OR > 1, *p* < 0.05 under IVW], while six (MYO9A, MTIF2, BAZ2B, NFKBIZ, OSGIN2, and RTTN) were considered protective (OR < 1, *p* < 0.05 in IVW) (Table S6). Scatter plots demonstrated positive correlations between the aforementioned risk genes and MN, and negative correlations between the protective genes and MN (Figure S1). A forest plot analysis revealed that the IVW estimates were consistently greater than zero for the eight risk genes and less than zero for the six protective genes (Figure S2). Funnel plot symmetry supported the assumption of random allele assortment, consistent with Mendel’s second law (Figure S3), and the selected IVs displayed a largely symmetrical and numerically balanced distribution, affirming compliance with core MR assumptions. Sensitivity analyses were undertaken to validate the robustness of the causal inferences. Heterogeneity testing showed Q-statistic *p*-values > 0.05 for 13 of the 14 exposures, supporting the use of a fixed-effects IVW model. For BAZ2B, where heterogeneity was indicated (*p* < 0.05), a random-effects IVW model was applied (Table S7). Horizontal pleiotropy tests yielded non-significant results (*p* > 0.05), indicating minimal bias from confounding factors. The final gene set retained for downstream analysis included MYO9A, MTIF2, USP40, ZFAND1, FNBP4, BAZ2B, GPR18, TIGD7, NFKBIZ, OSGIN2, NOC3L, RTTN, WDR73, and PPIL3 (Table S8). LOO analyses confirmed that the exclusion of individual SNPs did not significantly alter the causal estimates, underscoring the stability of the findings (Figure S4). Furthermore, the Steiger directional test indicated that all 14 gene–trait associations satisfied the directional criterion (*p* < 0.05), reinforcing the validity of the inferred causal relationships (Table S9). These 14 MD-DEGs were thus prioritized as candidate signature genes with potential causal involvement in MN pathogenesis.

### 3.4. Identification of Key Genes and Construction of Nomogram

**Figure 4:**
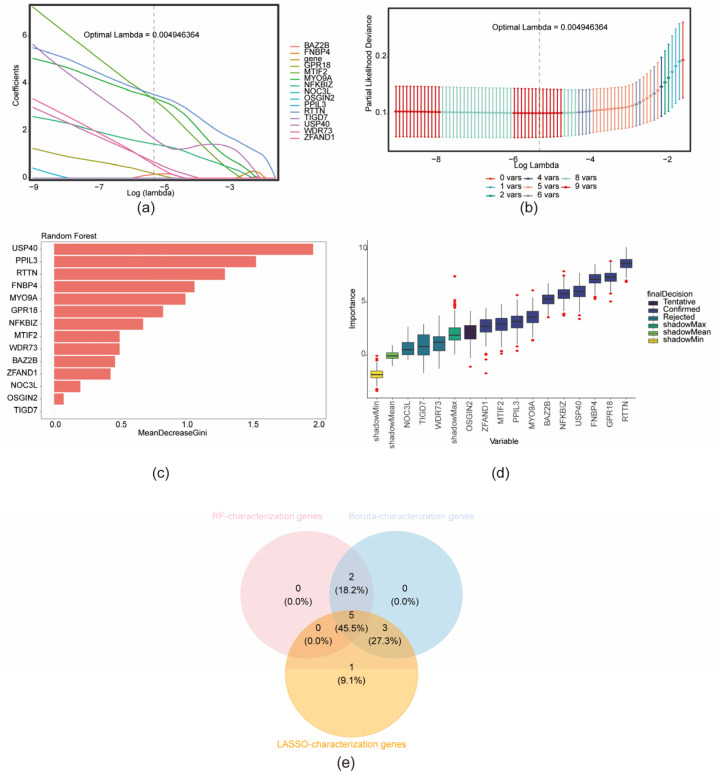
Identification of 5 characterization genes and construction of machine-learning. (**a**) Selection of the optimal lambda value for LASSO. (**b**) LASSO coefficient penalty plot. (**c**) Random forest model. (**d**) Boruta algorithm gene importance ranking plot. (**e**) Identification of 5 feature genes.

**Figure 5:**
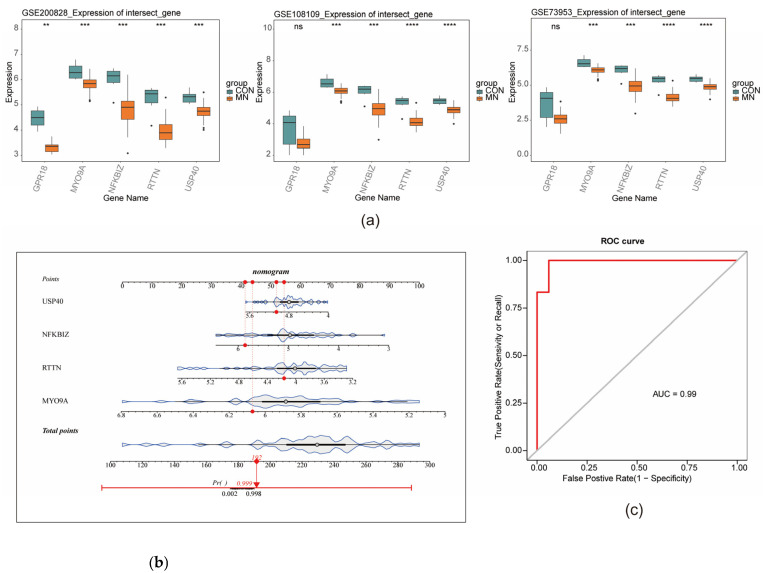
Identification of key genes and construction of nomogram model. (**a**) Expression differences of feature genes in the training and validation sets. (**b**) Nomogram model was constructed based on key genes. (**c**) ROC curve evaluation of the predictive performance of the nomogram model was performed. ** represents *p* < 0.01, *** signifies *p* < 0.001, **** denotes *p* < 0.0001. “ns” represents no significant difference.

Based on the previously identified candidate characterization genes, further screening was conducted using multiple machine-learning algorithms. LASSO regression was applied, retaining genes corresponding to the minimum mean cross-validation error (λ_min = 0.004946), resulting in the identification of nine LASSO-derived characterization genes (Figure 4a,b). Additionally, the RF algorithm identified seven RF-characterization genes (Figure 4c), while the Boruta algorithm selected ten Boruta-characterization genes (Figure 4d). The intersection of gene sets obtained from the three algorithms yielded five consensus characterization genes: MYO9A, USP40, GPR18, NFKBIZ, and RTTN (Figure 4e). Expression validation of these five genes was performed using three datasets—GSE200828, GSE73953, and GSE108109—by comparing expression levels between the MN and control (CON) groups. Genes consistently exhibiting a significantly lower expression in the MN group across all datasets (*p* < 0.05) were considered key characterization genes. This analysis identified four genes: MYO9A, NFKBIZ, RTTN, and USP40 (Figure 5a). A nomogram incorporating these four key genes was constructed based on the GSE200828 dataset to estimate individual risk of developing MN. Increased nomogram scores corresponded to elevated predicted risk (Figure 5b). The model demonstrated an excellent predictive accuracy, with an AUC of 1.0 (Figure 5c).

### 3.5. Subcellular and Chromosomal Localization and Functional Enrichment Analysis

**Figure 6:**
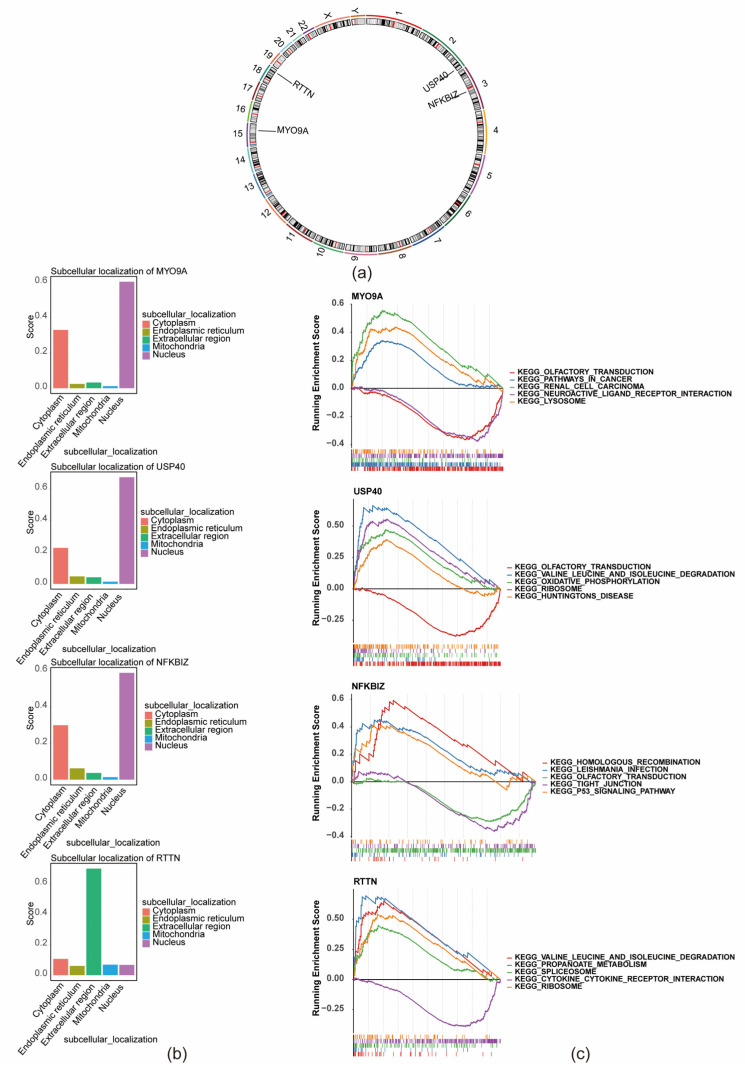
Chromosomal and subcellular localization and functional enrichment of key genes. (**a**) Chromosomal localization of key genes. (**b**) Subcellular localization of key cells. (**c**) Functional pathway analysis of key genes.

Chromosomal mapping revealed that RTTN is located on chromosome 18, MYO9A on chromosome 15, USP40 on chromosome 2, and NFKBIZ on chromosome 3 (Figure 6a). Subcellular localization analysis indicated that MYO9A, NFKBIZ, and USP40 are predominantly expressed in the nucleus, whereas RTTN is primarily localized to the extracellular region (Figure 6b). GSEA showed that MYO9A was enriched in 68 pathways, with key pathways including olfactory transduction and neuroactive ligand–receptor interaction. USP40 was enriched in 38 pathways, including olfactory transduction, valine, leucine, and isoleucine degradation, and oxidative phosphorylation. NFKBIZ was enriched in six pathways, notably homologous recombination, leishmaniasis infection, and olfactory transduction. RTTN was enriched in 73 pathways, with primary associations involving valine, leucine, and isoleucine degradation, propanoate metabolism, and spliceosome pathways (Figure 6c).

### 3.6. Immune Microenvironment in MN

**Figure 7:**
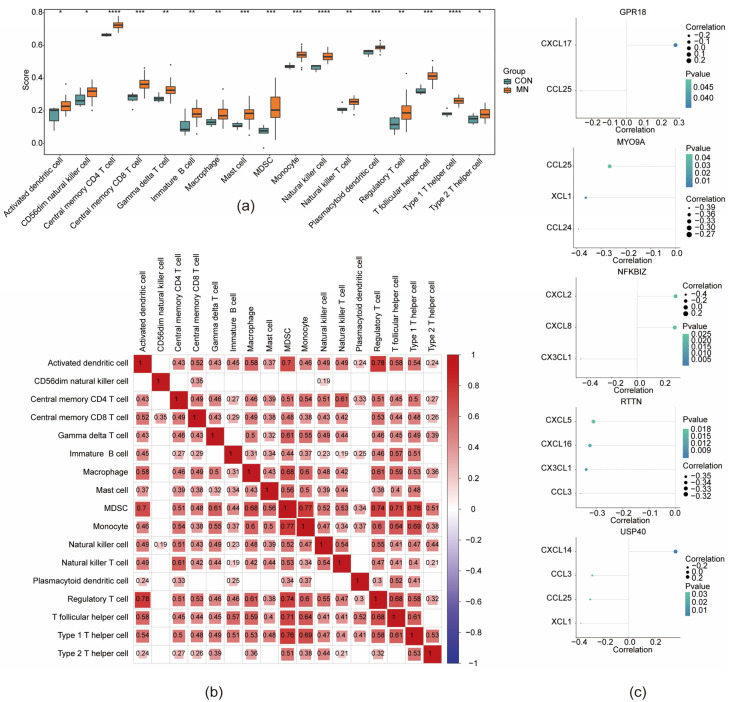
The immune microenvironment of membranous nephropathy. (**a**) Expression differences of 17 immune cell types between the disease and control groups. (**b**) Correlation heatmap of the 17 immune cell types. (**c**) Bubble plot of the correlation between key genes and different immune factors. * indicates that *p* < 0.05, ** represents *p* < 0.01, *** signifies *p* < 0.001, **** denotes *p* < 0.0001.

Immune cell infiltration analysis was conducted using the GSE200828 dataset to derive immune cell infiltration scores. Samples demonstrating statistically significant immune cell enrichment (*p* < 0.05) were included for further analysis, while samples lacking enrichment were excluded. Boxplot visualization revealed differential expression profiles across 17 immune cell types between the MN and CON groups. These included activated dendritic cells, CD56^dim^ natural killer cells, central memory CD4^+^ and CD8^+^ T cells, γδ T cells, immature B cells, macrophages, mast cells, myeloid-derived suppressor cells (MDSCs), monocytes, natural killer cells, natural killer T cells, plasmacytoid dendritic cells, regulatory T cells (Tregs), T follicular helper cells, and type 1 and type 2 T helper cells (Figure 7a). Correlation analysis within MN samples revealed that regulatory T cells and activated dendritic cells exhibited the strongest positive interrelationship among the 17 immune cell types, with a correlation coefficient of 0.78 (*p* < 0.05) (Figure 7b). Further investigation into the associations between key genes and immune modulators identified several significant correlations: MYO9A was negatively correlated with checkpoint gene CCL24 (cor = −0.36, *p* < 0.05); NFKBIZ showed the strongest positive correlation with CXCL2 (cor = 0.30, *p* = 0.02); RTTN exhibited the strongest negative correlation with CXCL5 (cor = −0.31, *p* = 0.02); and USP40 demonstrated a positive correlation with CXCL14 (cor = 0.36, *p* = 0.01) (Figure 7c, Table S10).

### 3.7. Acquisition of Regulatory Relationships

**Figure 8:**
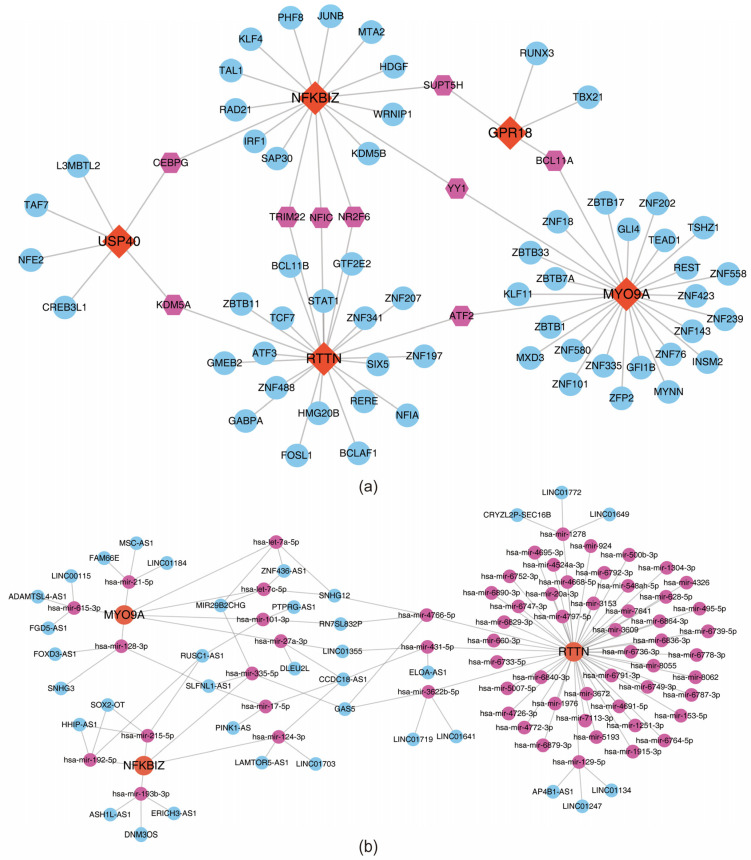
Molecular regulatory network of key genes. (**a**) Key gene–TF network diagram. (**b**) lncRNA–miRNA–mRNA network diagram.

The TF–key gene regulatory network incorporated 4 key genes and 65 TFs. RTTN and MYO9A were found to co-regulate ATF2; RTTN and USP40 co-regulated KDM5A; and RTTN and NFKBIZ co-regulated NFIC (Figure 8a). Additionally, the integrated mRNA–miRNA–lncRNA regulatory network elucidated downstream regulatory relationships: RTTN modulated ELOA-AS1 via hsa-miR-431-5p, and MYO9A regulated SNHG12 via hsa-let-7c-5p, among others (Figure 8b).

### 3.8. Identification of Key Cell

**Figure 9:**
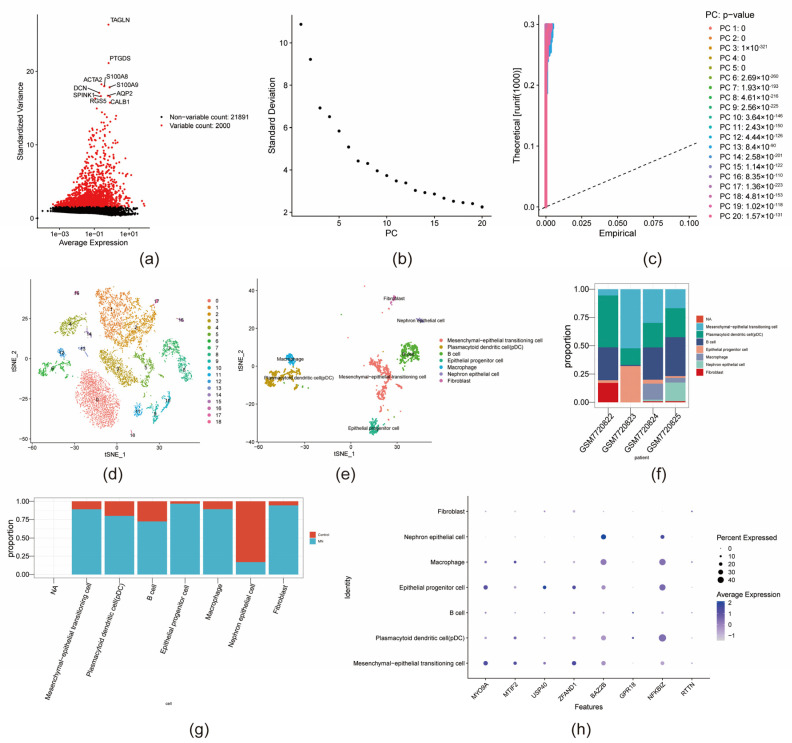
Single-cell dataset analysis of membranous nephropathy. (**a**) Screening of highly variable genes. (**b**) Scree plot of the top 20 principal components. (**c**) Jackstraw permutation test plot. (**d**) Unsupervised clustering analysis plot. (**e**) Seven cell types identified. (**f**) Differences in the proportions of cell clusters in MN and control groups. (**g**) Expression patterns of seven cell clusters in MN and control groups. (**h**) Bubble plot of key gene expression in each cell cluster.

Feature RNA quantity metrics and raw gene counts were assessed before and after QC (Figure S5a,b). Following QC, 2000 highly variable genes were selected for downstream analyses (Figure 9a). PCA was performed on the transcriptomic dataset, with the top 20 principal components retained for clustering and visualization (Figure 9b,c). Subsequent unsupervised clustering resulted in the identification of 19 distinct clusters, which were annotated into seven cell types: mesenchymal–epithelial transitioning cells, plasmacytoid dendritic cells, B cells, epithelial progenitor cells, macrophages, nephron epithelial cells, and fibroblasts (Figure 9e). The proportional distribution of these annotated cell types within the GSE241302 dataset is depicted in Figure 9g. Differential cell-type expression patterns between the MN and control (CON) groups are presented in Figure 9f. NFKBIZ was found to be expressed in nephron epithelial cells, while MYO9A, RTTN, and USP40 showed no obvious expression signals in this cell type (Figure S6). Spatial mapping of key gene expression across the seven cell types (Figure 9h) revealed that NFKBIZ exhibited predominant expression in mesenchymal–epithelial transitioning cells and macrophages, whereas MYO9A was mainly expressed in mesenchymal–epithelial transitioning cells and epithelial progenitor cells. All four key genes demonstrated heightened expression within mesenchymal–epithelial transitioning cells when comparing MN to CON samples, thereby designating this population as a critical cell type in MN pathogenesis.

### 3.9. Communication Network and Pseudotime Analysis

**Figure 10:**
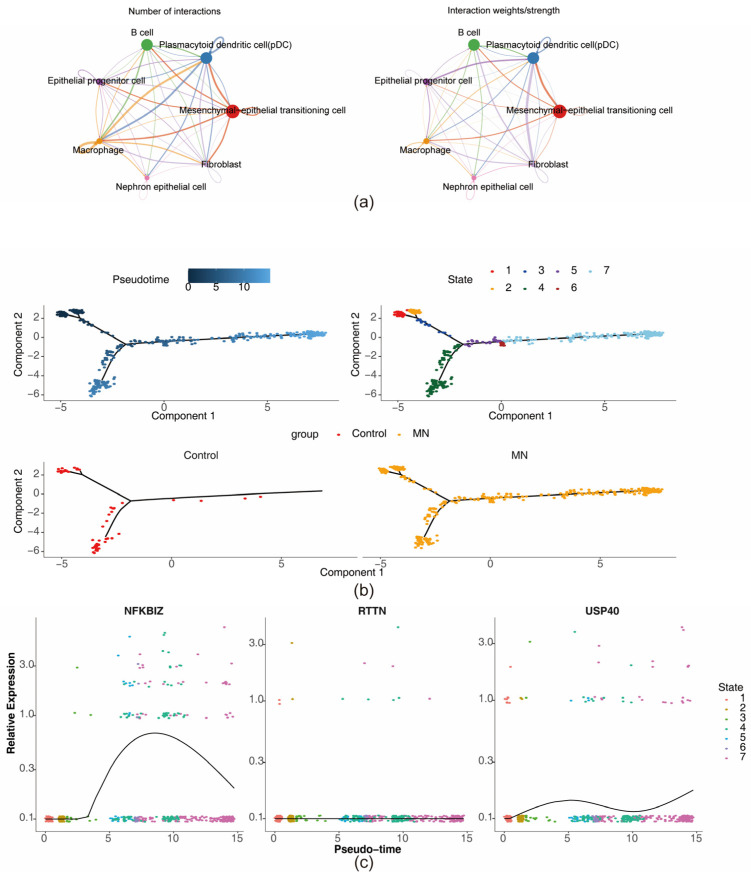
Cell communication network and pseudotime analysis. (**a**) The communication network between cells. Different colors represent different cell types, and the thickness of the lines represents the strength of cell–cell interactions. The thicker the line, the stronger the interaction. (**b**) Developmental trajectory of key cells. The top-left plot shows the differentiation timeline of cells, where deeper blue indicates earlier differentiation. The top-right plot illustrates seven different differentiation states of cells, with each state marked by a different color, and red represents the earliest differentiation type. The bottom-left plot shows the differentiation trajectory of key cells in the control samples. The bottom-right plot illustrates the differentiation trajectory of key cells in the MN samples. (**c**) Expression patterns of key genes in the seven differentiation states of key cells.

Cell–cell communication analysis indicated the strongest interaction strength between mesenchymal–epithelial transitioning cells and plasmacytoid dendritic cells (Figure 10a and Figure S7). Ligand–receptor interaction mapping further revealed that, while numerous intercellular connections existed, the most robust interaction occurred between fibroblasts and plasmacytoid dendritic cells. The highest interaction frequency, however, was observed in macrophage–macrophage pairings (Figure S8). Pseudotime trajectory analysis, anchored on mesenchymal–epithelial transitioning cells, was used to infer dynamic gene expression changes during cell differentiation. The expression profile of USP40 demonstrated an initial increase, followed by a decrease and a subsequent gradual upregulation over the pseudotime course. NFKBIZ expression showed a rapid increase in early pseudotime stages, followed by downregulation. In contrast, RTTN expression remained relatively unchanged (Figure 10b,c).

### 3.10. Validation of the Expression of Key Genes

**Figure 11:**
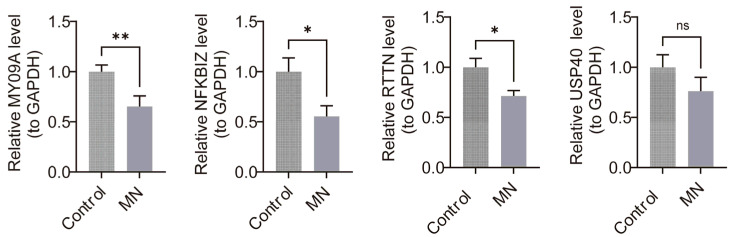
RT-qPCR validation of key genes. * indicates that *p* < 0.05, ** represents *p* < 0.01, “ns” represents no significant difference.

Validation of the transcriptomic findings was conducted through RT-qPCR to quantify MYO9A, NFKBIZ, RTTN, and USP40 expression levels in MN and control groups (Figure 11). The results confirmed a statistically significant downregulation of all four genes in the MN group relative to controls (*p* < 0.05).

## 4. Discussion

Membranous nephropathy (MN) constitutes a leading etiology of adult-onset nephrotic syndrome, where current therapeutic approaches, including immunosuppressive regimens, frequently fail to address either the underlying molecular pathogenesis or the persistent risk of disease recurrence [45]. Notably, dysregulation of mitochondrial dynamics (MD) disrupts cellular bioenergetic homeostasis, driving aberrant cellular fate determination through compromised mitochondrial quality control. In the renal tubular compartment, MD abnormalities may induce tubular epithelial atrophy, inflammatory cascades, and programmed cell death—pathobiological processes that collectively propagate renal disease progression [46]. Nevertheless, the precise mechanistic involvement of MD in MN pathogenesis remains incompletely characterized. To elucidate the molecular mechanisms underpinning MN, this study investigated the involvement of MD in disease pathogenesis. Transcriptomic datasets from public repositories were integrated with MR and machine-learning methodologies to identify MD-associated genes implicated in MN. Four candidate genes—MYO9A, NFKBIZ, RTTN, and USP40—were suggested to be involved in the pathological process of MN. Additionally, scRNA-seq analysis identified mesenchymal–epithelial transitioning cells as pivotal in the disease microenvironment. This comprehensive molecular characterization advances our current understanding of MN pathophysiology and lays a foundation for the development of targeted therapeutic interventions aimed at improving clinical outcomes.

Myosin IXA (MYO9A), a member of the myosin superfamily ubiquitously expressed in eukaryotic cells, exhibits low-level constitutive expression in renal tubular epithelial cells (particularly within proximal tubules and collecting ducts), while demonstrating significantly higher expression in renal interstitial fibroblasts [46]. This motor protein primarily modulates intracellular cytoskeletal architecture and dynamic remodeling, facilitating cellular migration and proliferative responses that contribute to progressive renal fibrogenesis [47]. Mechanistically, MYO9A may orchestrate inflammatory amplification in the tubular epithelium by regulating the nuclear translocation kinetics of the NF-κB signaling axis, thereby indirectly modulating secretory profiles of pro-inflammatory cytokines such as interleukin-6 (IL-6) [47]. Experimental models have demonstrated that MYO9A deficiency in mice results in proximal tubular dilation and interstitial fibrosis, underscoring its regulatory function in tubular physiology [48]. MN is frequently accompanied by tubular-interstitial pathological alterations and fibrosis. Moreover, MYO9A has emerged as a candidate gene for focal segmental glomerulosclerosis, implicated in the regulation of podocyte adhesion and migration [49]. Although the precise involvement of MYO9A in MN pathogenesis remains undefined, preliminary findings suggest that the dysregulation of MYO9A may intensify tubular injury and compromise podocyte dynamics, offering novel therapeutic avenues for MN. Nuclear factor kappa B inhibitor zeta (NFKBIZ), a member of the IκB family, negatively regulates NF-κB signaling and significantly influences immune modulation and inflammatory responses [50]. NFKBIZ demonstrates compartmentalized expression within the nephron, with predominant localization observed in renal tubular epithelial cells (particularly enriched in proximal tubular segments) and glomerular mesangial cells, while exhibiting comparatively weaker expression profiles in renal endothelial cells [51,52]. Inhibition of the NF-κB pathway and the NLRP3 inflammasome has been shown to ameliorate MN manifestations in rat models, as reported by Liu et al. [53]. Additionally, phospholipase A2 group XII B (PLA2G12B) promotes MN pathogenesis by activating the NF-κB pathway and facilitating arachidonic acid metabolism [54]. Although direct evidence linking PLA2G12B to MN is lacking, its regulatory influence on NF-κB signaling suggests a potential contributory role in disease progression. Rotatin (RTTN), encoding a large centrosomal protein, is known to be mutated in cerebral malformation syndromes characterized by conditions such as multiple cerebellar gyri, microcephaly, primordial dwarfism, and epileptic seizures [55]. To date, no association between RTTN and renal pathology has been established. Ubiquitin-specific protease 40 (USP40), emerging as a novel deubiquitinating enzyme (DUB) within the USP family [54], manifests pan-tubular expression patterns with particular abundance in renal tubular epithelial cells (proximal and distal convoluted tubules), while maintaining moderate expression levels in renal endothelial cells and glomerular mesangial compartments [56]. In zebrafish models, Usp40 knockout induces glomerular disorganization, disruption of endothelial cell junctions, and effacement of podocyte foot processes [57]. Furthermore, USP40 has been reported to deubiquitinate HINT1 and stabilize p53 in the context of podocyte injury [58], implying a possible mechanistic role in MN by modulating podocyte integrity. Notably, this study marks the first identification of four key genes potentially implicated in MN. These genes present promising therapeutic targets, and ongoing investigations aim to further delineate their pathogenic roles and underlying molecular mechanisms.

MYO9A demonstrates significant enrichment within lysosome-related pathways, particularly involving autophagic flux regulation and endosomal–lysosomal trafficking networks. Evidence suggests that in idiopathic MN, inhibition of the lysosome-dependent autophagic process can alleviate podocyte injury induced by the terminal complement complex C5b-9, highlighting the protective role of lysosomal function in glomerular homeostasis [59]. Furthermore, comparative studies have demonstrated a more-than-twofold increase in the expression of lysosomal membrane protein-2 (LIMP-2) in the glomeruli of patients with idiopathic MN relative to healthy controls [60]. Collectively, these findings underscore the critical involvement of lysosomes in MN pathophysiology, suggesting that MYO9A may contribute to disease progression through its modulation of lysosomal activity.

Aberrations in B cell biology are particularly prominent in primary MN (PMN), encompassing disruptions in B cell subset composition and functional regulation. B cells execute distinct immunological roles at various disease stages, including the presentation of self-antigens, activation of effector T cells, and production of pathogenic autoantibodies [61]. According to Chen Lu et al., patients with MN exhibit elevated frequencies of B cells, follicular helper T (Tfh) cells, and follicular regulatory T (Tfr) cells in peripheral blood compared to healthy individuals. Notably, Tfr cells from patients with MN display decreased CTLA-4 expression and increased PD-1 expression, suggesting an impaired immunoregulatory capacity, which may exacerbate disease pathology [62]. Additionally, Liu et al. highlighted the pathogenic relevance of cTfh cell subset imbalances in MN. Specifically, cTfh2 cells promote B cell differentiation via IL-21 secretion, potentially serving as a biomarker for disease activity and progression [60]. These insights emphasize the importance of immune cell dysregulation in MN and support the value of further investigations into the roles of specific lymphocyte subsets in its pathogenesis.

These cells possess the capacity to revert from a mesenchymal phenotype to an epithelial phenotype through a process known as mesenchymal–epithelial transition (MET), which is mechanistically the opposite of epithelial–mesenchymal transition (EMT). MET plays a pivotal role in embryogenesis, tissue regeneration, and tumor metastasis [63,64]. In renal physiology, nephron endowment—and consequently, kidney function—is closely linked to the formation of renal tubules, a process fundamentally dependent on MET. Disruption of this process has been associated with congenital anomalies of the kidney as well as the progression of chronic kidney disease in adults. Experimental findings indicate that lithium ions can induce MET in human renal mesenchymal cells, a transition characterized by the upregulation of epithelial and tubular markers and concurrent downregulation of renal progenitor and mesenchymal markers [65]. Additionally, prokineticin receptor 1 (PKR1) has been shown to regulate renal tubulogenesis and glomerulogenesis during kidney development by modulating MET, cellular proliferation, and apoptosis [66]. Beyond developmental biology, MET also holds relevance in oncology. A systematic review and meta-analysis has synthesized current evidence regarding the therapeutic efficacy and safety of MET inhibitors in patients with advanced papillary renal cell carcinoma. However, due to a high incidence of treatment-related adverse events, clinical application warrants careful consideration. Further large-scale, multiethnic studies are required to validate the therapeutic efficacy and safety profile of diverse MET inhibitors across patient populations.

The pathogenesis of membranous nephropathy (MN) is intricately associated with the dysregulated expression of core regulatory genes including MYO9A, NFKBIZ, RTTN, and USP40, which collectively demonstrate multidimensional clinical relevance spanning disease progression monitoring, prognostic stratification, and therapeutic target development. Mechanistically, these genes act as follows:

MYO9A downregulation exacerbates renal interstitial fibrosis and accelerates functional deterioration [49].

NFKBIZ-mediated suppression of the NF-κB signaling axis mitigates inflammatory cascades [67].

RTTN ensures preservation of podocyte cytoarchitectural integrity [68].

USP40 safeguards mitochondrial homeostasis in renal tubular networks [69].

The machine-learning-driven nomogram model integrating these biomarkers achieves an AUC of 0.99, demonstrating exceptional diagnostic efficacy that surpasses conventional biomarkers [70]. These molecular targets not only serve as dual-purpose diagnostic-prognostic indicators, but also pioneer novel therapeutic dimensions through precision interventions targeting the following:

Fibrotic reprogramming pathways;

Inflammatory signal amplification nodes;

Podocytopathic injury mechanisms;

Mitochondrial bioenergetic dysfunction.

This multi-omics derived framework substantially enhances clinical translatability, offering stratified therapeutic strategies for MN precision medicine.

This investigation acknowledges several methodological constraints that warrant consideration. Primarily, the heavy reliance on bioinformatics predictions and computational modeling—without orthogonal functional validation through gene knockdown, overexpression, or pathway perturbation assays—may introduce interpretive bias, potentially obscuring comprehensive understanding of hub genes’ pathobiological roles in MN. Secondly, the absence of protein-level verification (via immunohistochemistry, Western blotting, or immunofluorescence) leaves critical gaps in characterizing the spatial expression patterns of target genes within MN renal tissues, while the lack of genetic manipulation studies precludes mechanistic insights into their regulatory effects on MN-associated phenotypes such as apoptotic cascades, migratory behavior, or secretory profiles.

Notably, the limited clinical sample size for RT-qPCR validation (particularly the statistically insignificant USP40 expression discrepancy despite GEO dataset indications) underscores potential Type II errors arising from inadequate statistical power. Furthermore, the unavailability of granular clinical parameters (e.g., proteinuria quantification, eGFR trajectories) in existing GEO datasets currently restricts progression correlation analyses.

To address these limitations, our phased research roadmap proposes the following.

Multi-modal protein validation: 

Systematic IHC/WB/IF characterization of hub gene expression gradients across renal microanatomical domains (podocytes, tubular epithelia, interstitial compartments).

Mechanistic interrogation:

In vitro CRISPR/Cas9-mediated gene editing combined with small-molecule inhibitors/RNAi in glomerular/tubular cell models;

In vivo phenotyping of MN progression markers (apoptotic indices, motility profiling, secretome analysis).

Clinical corroboration:

Expanded RT-qPCR validation cohorts with histopathological staging stratification;

Integration of matched clinical metadata (24 h proteinuria, dynamic eGFR) to decode the immune microenvironment–MN progression interplay.

Key enhancements:

Transition from predictive analytics to causal mechanism dissection;

Implementation of spatial omics approaches for microenvironmental mapping;

Development of therapeutic response prediction algorithms through machine-learning-augmented clinical datasets.

This iterative framework aims to transform observational associations into validated therapeutic targets, bridging the current chasm between computational discovery and clinical translation in MN research.

## 5. Conclusions

This study utilized publicly available transcriptomic datasets to identify four pivotal genes—MYO9A, NFKBIZ, RTTN, and USP40—that are potentially implicated in MD within the context of MN, thereby establishing a foundation for more comprehensive exploration of the underlying biological processes. Through robust bioinformatics analyses, meaningful associations between these candidate genes and MN have been delineated, contributing to an enhanced understanding of the disease’s molecular landscape.

## Data Availability

The datasets (GSE200828, GSE73953, and GSE108109) analyzed during the current study are available in the Gene Expression Omnibus (GEO) repository (https://www.ncbi.nlm.nih.gov/gds).

## Supplementary Materials

The following supporting information can be downloaded at https://www.mdpi.com/article/10.3390/biomedicines13061489/s1, Figure S1: Scatter plot of the correlation between exposure factors and outcomes; Figure S2: Forest plot of the diagnostic efficacy of exposure factors on outcomes; Figure S3: Funnel plot for MR randomization evaluation; Figure S4: Forest plot for sensitivity analysis by sequential exclusion; Figure S5: Feature RNA quantity metrics and raw gene counts were assessed before and after QC(a) scRNA-seq data of MN samples. nFeature_RNA: The number of genes detected in each cell, nCount_RNA: The gene expression count detected in each cell, percent.mt: Mitochondrial gene percentage. (b) Quality control of scRNA-seq data; Figure S6: The expression distribution of key genes in different cell types; Figure S7: communication network among the 7 cells; Figure S8: Potential interactions between cells. Different colors indicate the communication probability, with blue representing a lower probability and red representing a higher probability. Table S1: RT-qPCR primer sequences; Table S2: 211 BPs of MD-DEGs; Table S3: 35 CCs of MD-DEGs; Table S4: 51 MFs of MD-DEGs; Table S5: 10 KEGG pathways of MD-DEGs; Table S6: MR randomization analysis results of different exposure factors; Table S7: Heterogeneity test results of exposure factors; Table S8: Multilevel pleiotropy test results of exposure factors; Table S9: Steiger reversed causality test results of exposure factors; Table S10: Correlation between key genes and immune factors.

## Abbreviations

The following abbreviations are used in this manuscript:

MN	Membranous Nephropathy
MD	Mitochondrial Dynamics
MDGs	Mitochondrial Dynamics-Related Genes
scRNA-seq	Single-Cell RNA Sequencing
AUC	Area Under the Curve
RT-qPCR	Reverse-Transcription Quantitative PCR
GEO	Gene Expression Omnibus
DEGs	Differentially Expressed Genes
WGCNA	Weighted Gene Co-expression Network Analysis
GO	Gene Ontology
KEGG	Kyoto Encyclopedia of Genes and Genomes
PPI	Protein–Protein Interaction
SNPs	Single Nucleotide Polymorphisms
MR	Mendelian Randomization
IVW	Inverse Variance Weighted
LASSO	Least Absolute Shrinkage and Selection Operator
RF	Random Forest
ROC	Receiver Operating Characteristic
PCA	Principal Component Analysis
MET	Mesenchymal–Epithelial Transition
EMT	Epithelial–Mesenchymal Transition
TF	Transcription Factor
miRNA	MicroRNA
lncRNA	Long Non-coding RNA
Tfh	Follicular Helper T cells
Tfr	Follicular Regulatory T cells
PMN	Primary Membranous Nephropathy
CTLA-4	Cytotoxic T-Lymphocyte-Associated Protein 4
PD-1	Programmed Cell Death Protein 1
IL-21	Interleukin-21
PKR1	Pre-Kinase Receptor 1
