# Supplementary figures and images for: Identification Exploring the Mechanism and Clinical Validation of Mitochondrial Dynamics-Related Genes in Membranous Nephropathy Based on Mendelian Randomization Study and Bioinformatics Analysis
Source: Biomedicines. 2025 Jun 17;13(6):1489. doi: 10.3390/biomedicines13061489 (PMC12191289; doi:10.3390/biomedicines13061489)

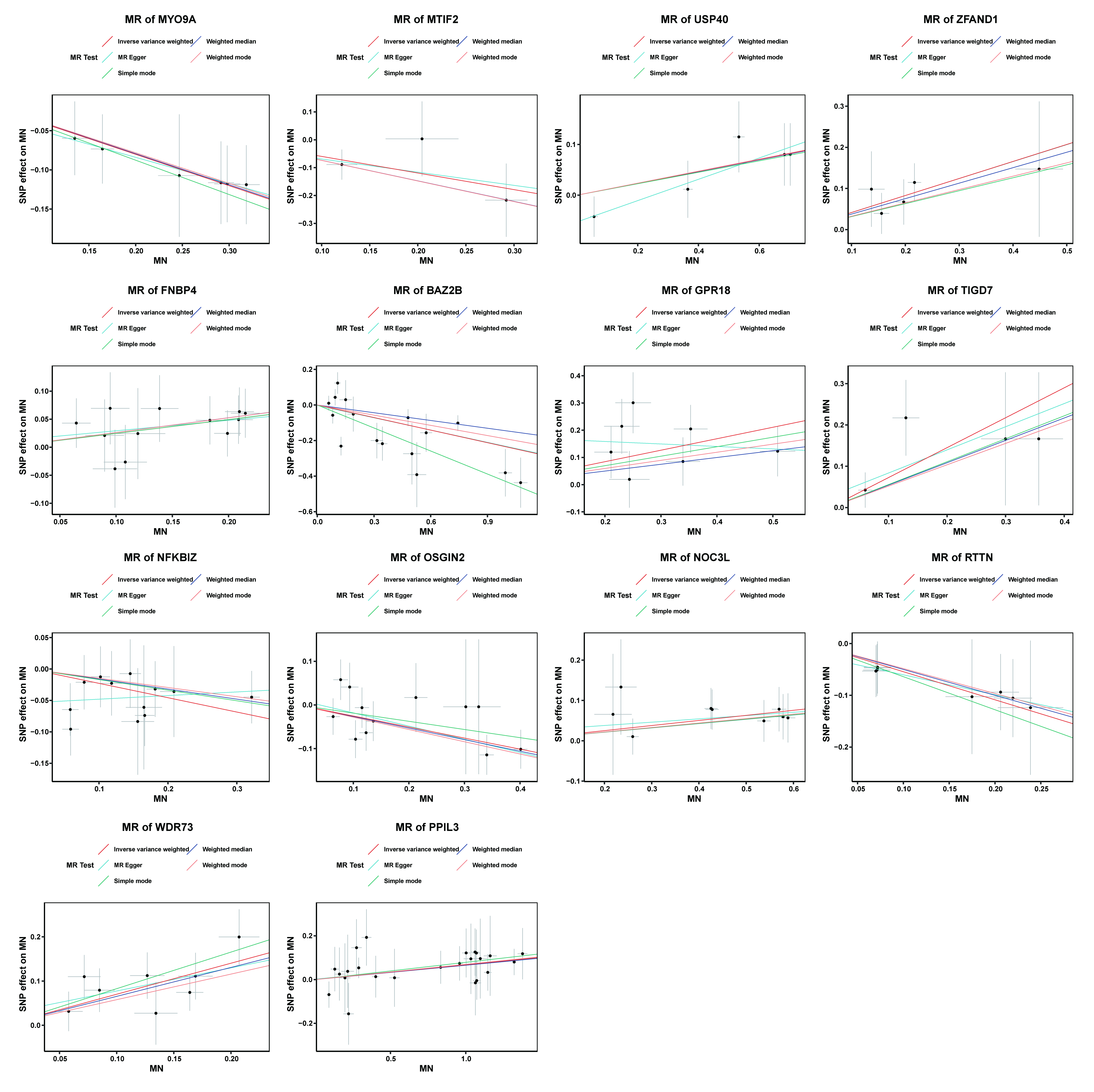

Supplement: Supplementary file 1 [file biomedicines-13-01489-s001.zip › Figure_S1.tif]

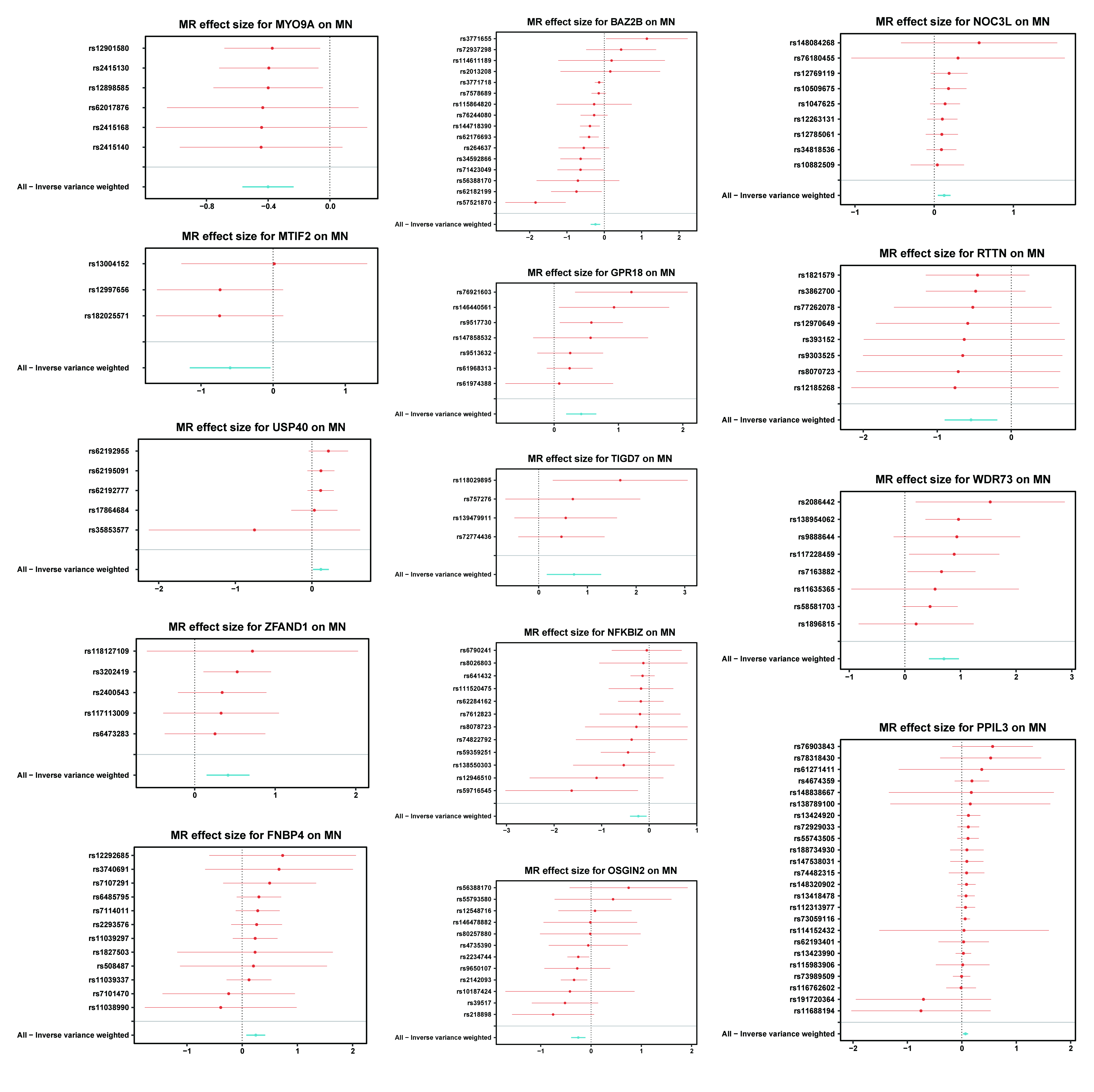

Supplement: Supplementary file 1 [file biomedicines-13-01489-s001.zip › Figure_S2.tif]

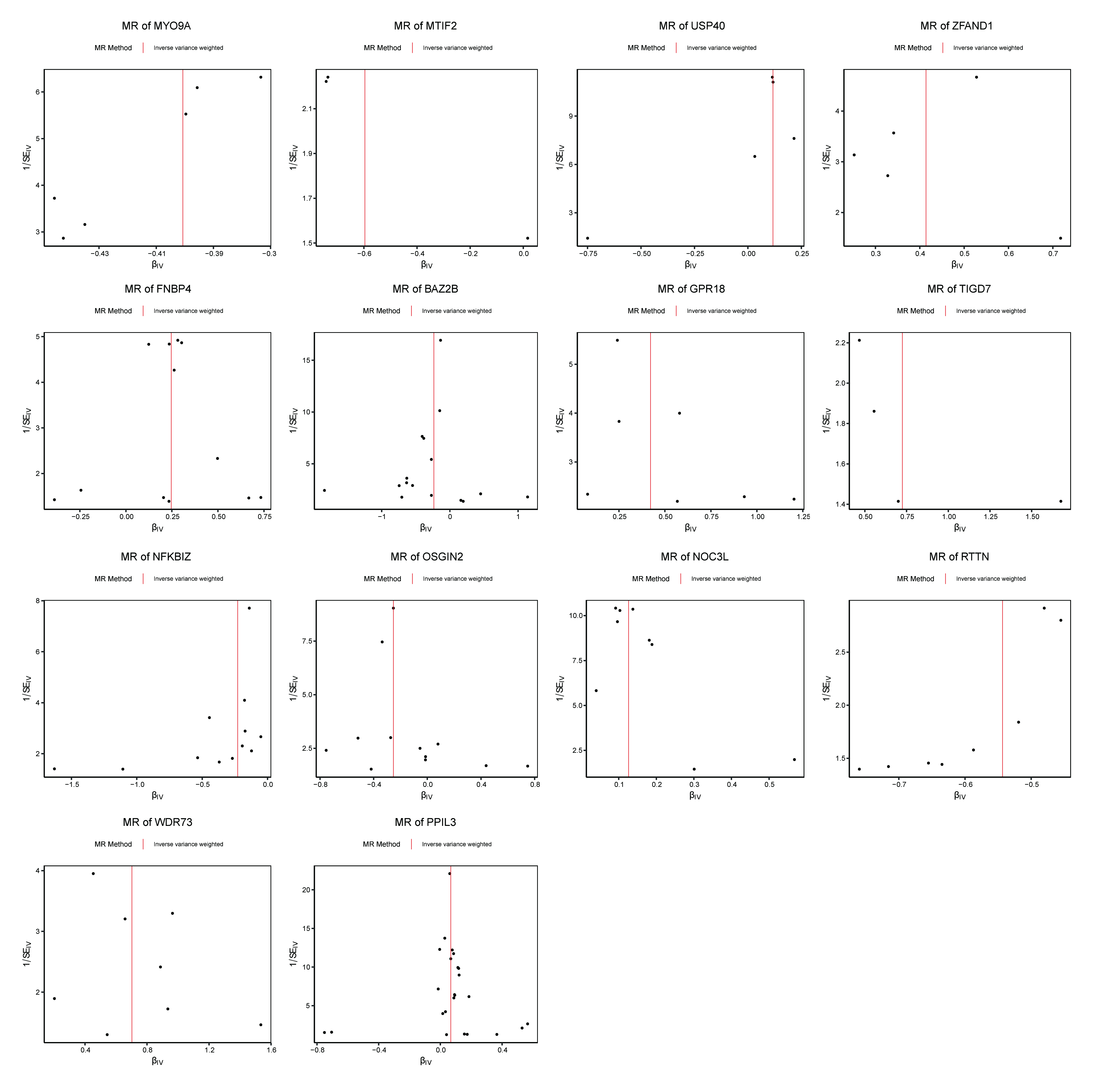

Supplement: Supplementary file 1 [file biomedicines-13-01489-s001.zip › Figure_S3.tif]

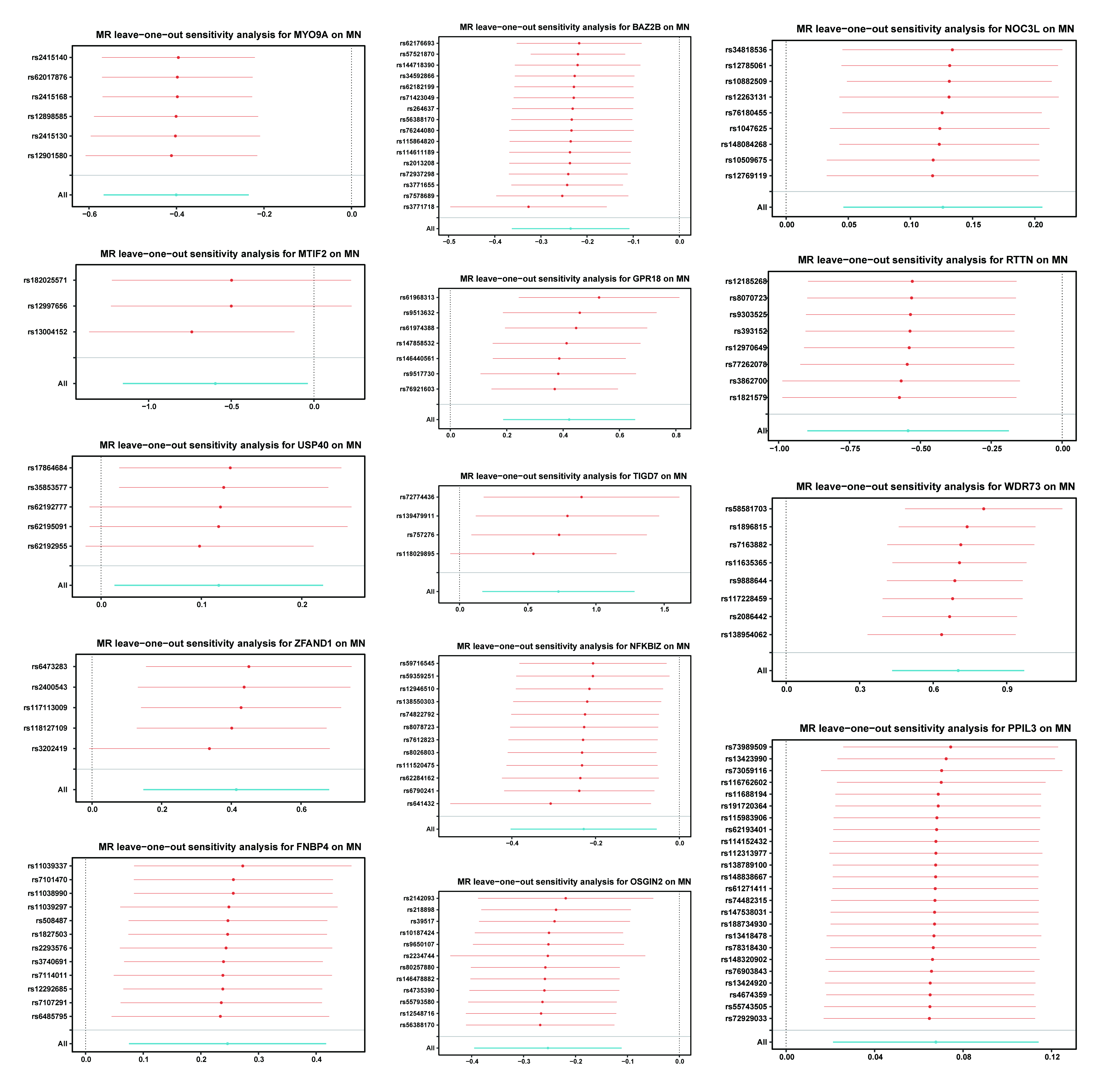

Supplement: Supplementary file 1 [file biomedicines-13-01489-s001.zip › Figure_S4.tif]

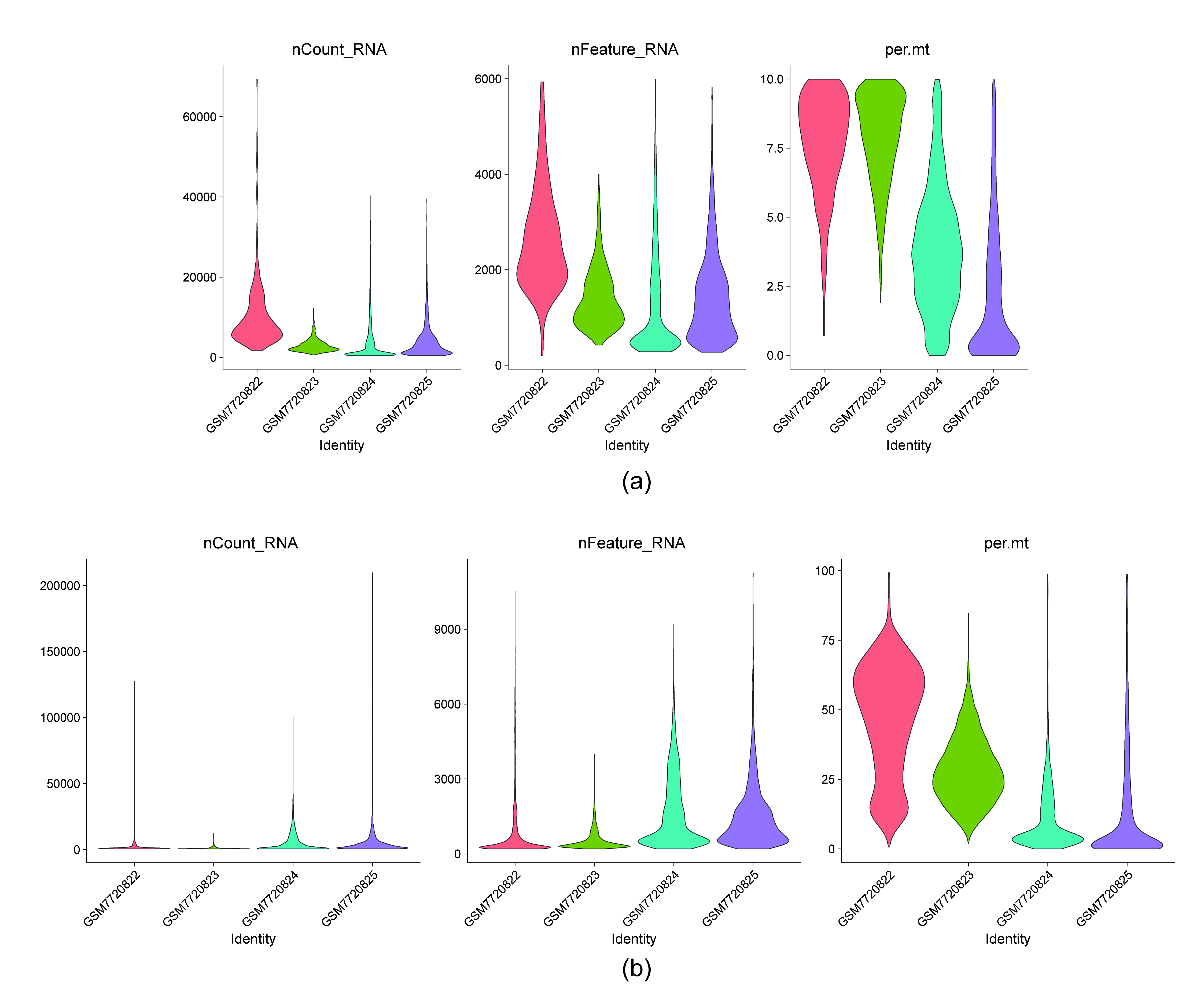

Supplement: Supplementary file 1 [file biomedicines-13-01489-s001.zip › Figure_S5.tif]

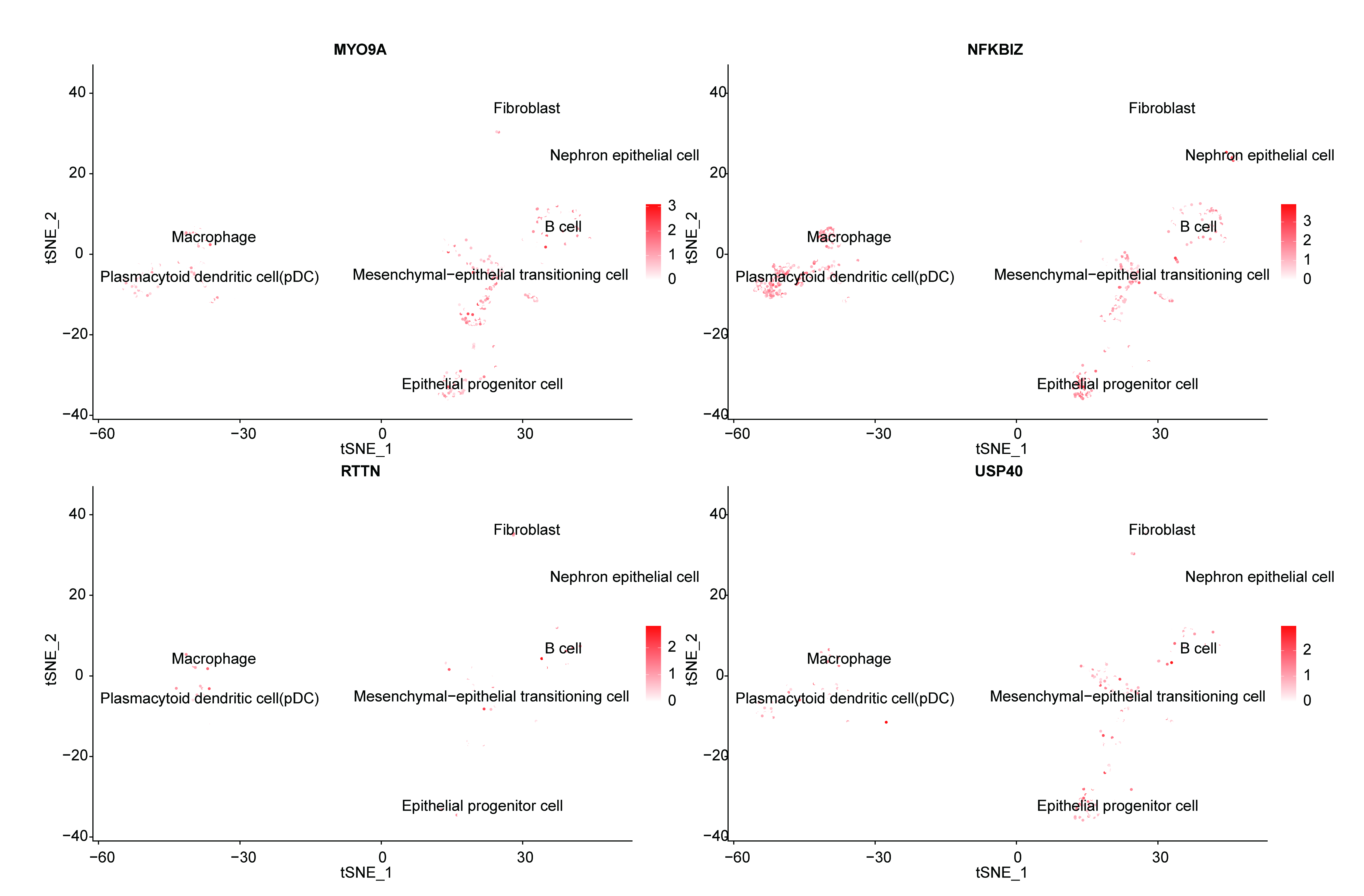

Supplement: Supplementary file 1 [file biomedicines-13-01489-s001.zip › Figure_S6.tif]

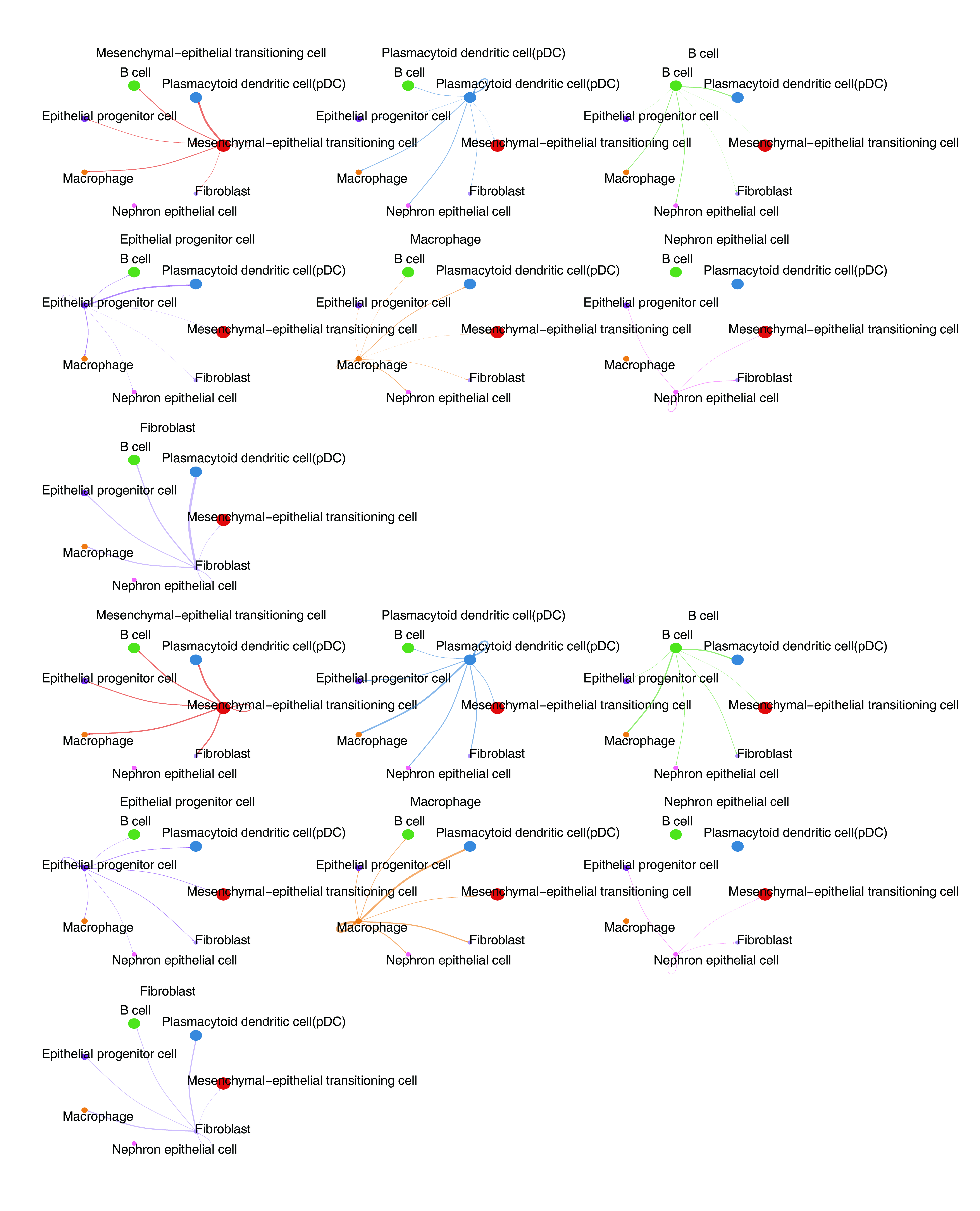

Supplement: Supplementary file 1 [file biomedicines-13-01489-s001.zip › Figure_S7.tif]

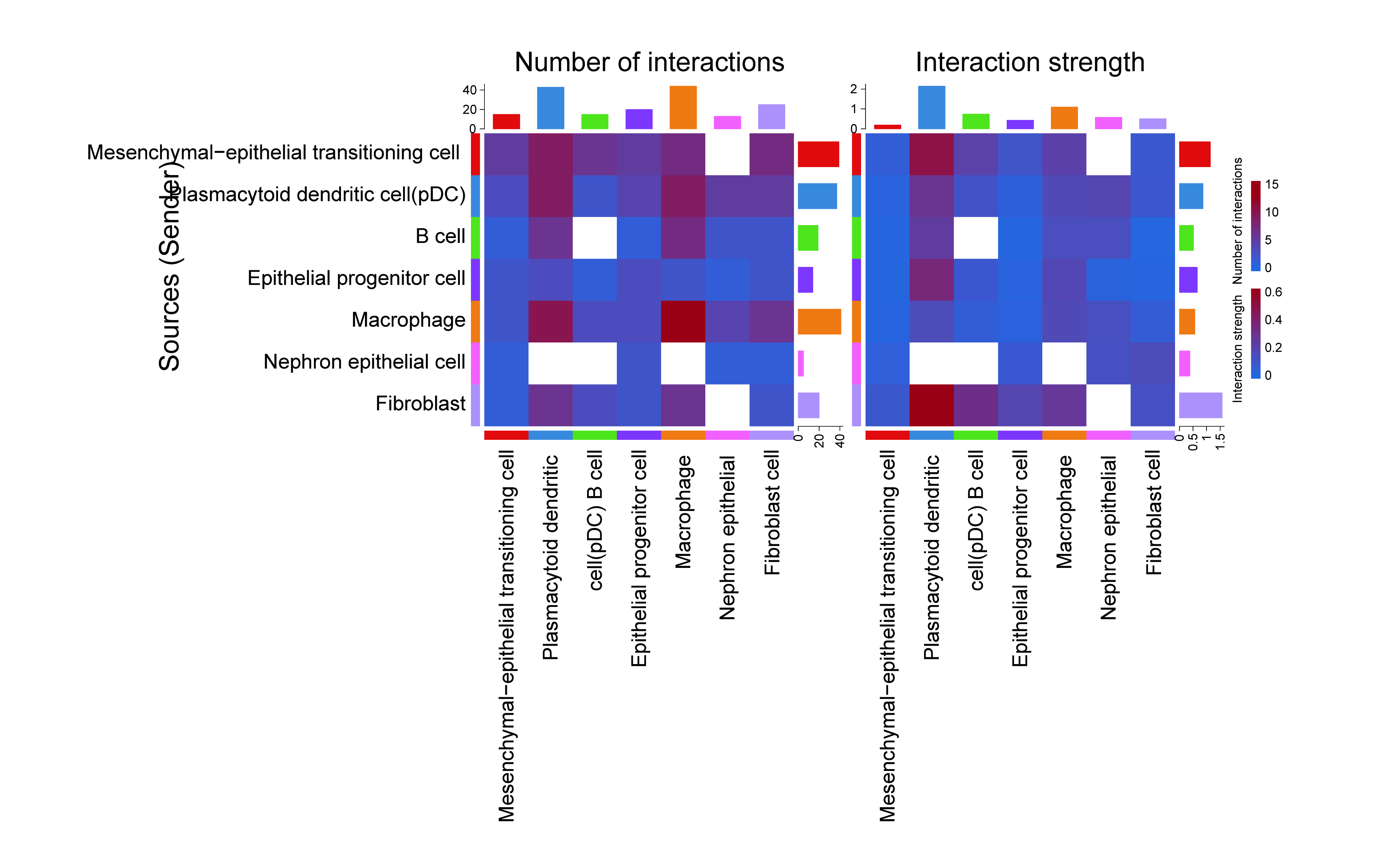

Supplement: Supplementary file 1 [file biomedicines-13-01489-s001.zip › Figure_S8.tif]
